# Constituents From the Flowers of *Viburnum macrocephalum* f. *macrocephalum* and Their *α*‐Amylase and *α*‐Glucosidase Inhibitory Activities

**DOI:** 10.1002/cbdv.71650

**Published:** 2026-08-31

**Authors:** Ya‐Jiao Chen, Hong‐Juan Zhou, Xue‐Jing Zhu, Jia Chen, Jian‐Hua Shao, Chun‐Chao Zhao

**Affiliations:** ^1^ College of Bioscience and Biotechnology Yangzhou University Yangzhou China

**Keywords:** phytochemical constituents, *Viburnum macrocephalum* f. *macrocephalum*, α‐amylase inhibition, α‐glucosidase inhibition

## Abstract

Phytochemical investigation of the flowers of *Viburnum macrocephalum* f. *macrocephalum* (VMM), a sterile horticultural form native to China, led to the isolation of two new cadinane‐type sesquiterpenes, viburetones A (**1**) and B (**2**), together with fifteen known compounds (**3**–**17**). Compounds **1** and **2** represent the first examples of this skeletal type from the genus *Viburnum*. The structures of the new compounds were elucidated by comprehensive analysis of their 1D and 2D NMR, HRESIMS, and ECD data. All isolates were evaluated for their inhibitory activities against *α*‐amylase and *α*‐glucosidase, key enzymes involved in carbohydrate metabolism. Among them, compound **1** exhibited inhibitory activities against *α*‐amylase (IC_50_ = 38.20 µM) and *α*‐glucosidase (IC_50_ = 24.15 µM), comparable to those of acarbose (IC_50_ = 39.19 and 25.73 µM, respectively). Molecular docking studies suggested that compound **1** bound to the active sites of both enzymes via hydrogen bonding and hydrophobic interactions.

## Graphical abstract

Two new cadinane‑type sesquiterpenes, viburetones A and B, were isolated from *Viburnum macrocephalum* flowers, marking the first cadinane skeletons in this genus. Viburetone A showed dual *α*‑amylase and *α*‑glucosidase inhibition comparable to acarbose, and docking clarified its binding. These flowers thus emerged as a promising source of such inhibitors for postprandial glycemic control.

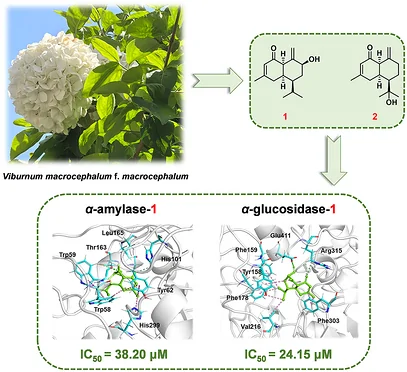

## 1 Introduction

The genus *Viburnum* (Adoxaceae) comprises over 230 species of shrubs and small trees distributed across temperate and subtropical regions. Species within this genus have long been used in traditional medicine for the treatment of fever, inflammation, and metabolic disorders [1, 2]. Phytochemical investigations have revealed that *Viburnum* spp. are rich sources of structurally diverse secondary metabolites, such as monoterpenes, diterpenoids, sesquiterpenes, and lignans, many of which exhibit significant biological activities, including antioxidant, anti‐inflammatory, antitumor, and neuroprotective effects [3, 4]. Notably, several *Viburnum* species have recently been reported to exhibit inhibitory activity against *α*‐amylase and *α*‐glucosidase [5, 6].

**FIGURE 1:**
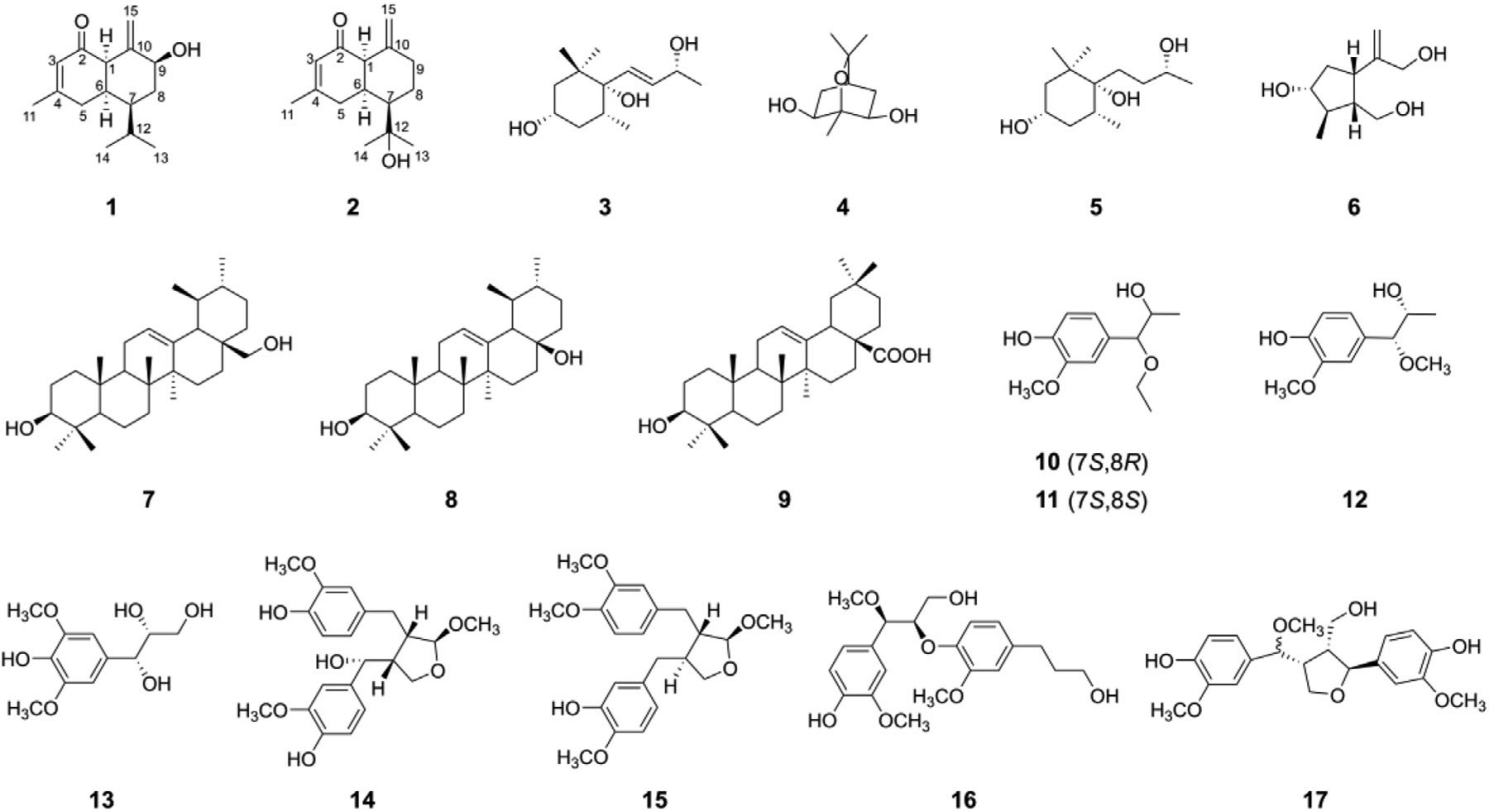
Chemical structures of compounds **1**‒**17**.

*Viburnum macrocephalum* f. *macrocephalum* (VMM) is a sterile horticultural form native to China, commonly known as “Muxiuqiu” or Chinese snowball viburnum, and is widely cultivated in Jiangsu, Zhejiang, Jiangxi, and Hebei provinces [7]. In traditional Chinese medicine, the flowers, stems, and leaves of VMM have been used for their dampness‐drying and antipruritic effects to treat scabies, tinea, and dampness‐induced pruritus [8]. Modern studies have focused on VMM flowers, finding that VMM flower extracts are rich in polyphenols and exhibit antioxidant, antibacterial, and antiviral activities [9, 10]. However, further phytochemical profiling and pharmacological evidence for VMM flowers remain scarce. As part of our ongoing search for *α*‐amylase and *α*‐glucosidase inhibitors from *Viburnum* species [5, 11], a comprehensive phytochemical investigation of VMM flowers was carried out. Herein, we report the isolation and structural elucidation of two new cadinane‐type sesquiterpenes, viburetones A (**1**) and B (**2**), together with fifteen known compounds (Figure 1), as well as the evaluation of their *α*‐amylase and *α*‐glucosidase inhibitory activities, followed by molecular docking analysis of compound **1**.

## 2 Results and Discussion

**FIGURE 2:**
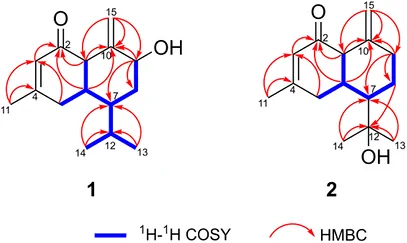
Key ^1^H‐^1^H COSY and HMBC correlations of compounds **1** and **2**.

**FIGURE 3:**
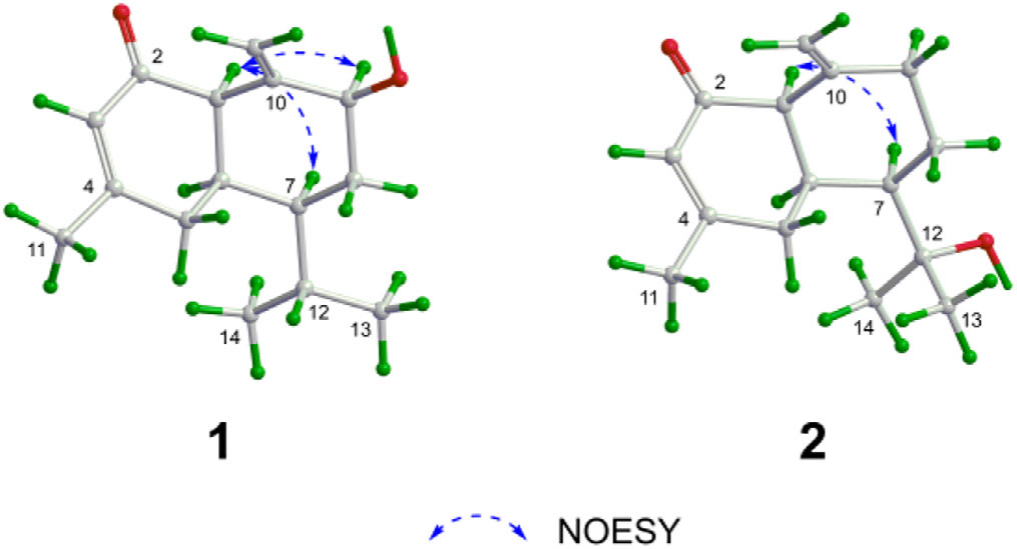
Key NOESY correlations of compounds **1** and **2**.

**FIGURE 4:**
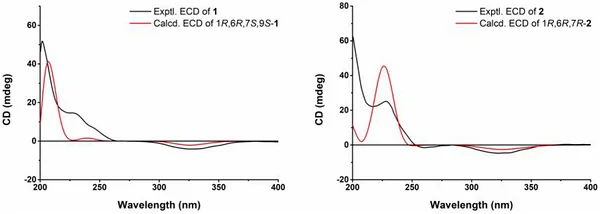
Calculated and experimental ECD spectra of compounds **1** and **2** in MeOH.

Compound **1**, a colorless oil, exhibited a sodium adduct molecular ion peak [M + Na]^+^ at *m/z* 257.1532 (calcd for C_15_H_22_O_2_Na^+^, 257.1512) in the HRESIMS spectrum, corresponding to the molecular formula C_15_H_22_O_2_ with five degrees of unsaturation (Figure S2). The IR spectrum (Figure S1) exhibited characteristic absorptions at 3407 cm^−1^ (hydroxyl group) and 1665 cm^−1^ (*α*,*β*‐unsaturated ketone). The ^1^H NMR spectrum of **1** (Table 1 and Figure S3) showed signals of three methyl groups [*δ*_H_: 1.96 (3H, s, H‐11), 0.94 (3H, d, *J* = 6.4 Hz, H‐13), and 0.95 (3H, d, *J* = 6.4 Hz, H‐14)], three methylenes [*δ*_H_: 2.10 (1H, overlapped, H‐5a) and 1.94 (1H, dd, *J* = 13.2, 4.2 Hz, H‐5b); 2.10 (1H, overlapped, H‐8a) and 1.47 (1H, m, H‐8b); 5.04 (1H, d, *J* = 1.9 Hz, H‐15a), and 4.52 (1H, d, *J* = 1.9 Hz, H‐15b)], and six methine protons [*δ*_H_: 3.40 (1H, d, *J* = 4.2 Hz, H‐1), 5.92 (1H, s, H‐3), 2.56 (1H, m, H‐6), 1.73 (1H, m, H‐7), 4.38 (1H, t, *J* = 3.0 Hz, H‐9), and 1.43 (1H, m, H‐12)]. The ^13^C NMR (Table 1 and Figure S4) and HSQC spectra (Figure S5) revealed 15 carbon resonances, including three methyls [*δ*_C_: 24.8 (C‐11), 20.9 (C‐13), and 21.4 (C‐14)], three methylenes [*δ*_C_: 27.3 (C‐5), 33.6 (C‐8), and 112.2 (C‐15)], six methines [*δ*_C_: 49.8 (C‐1), 126.2 (C‐3), 38.2 (C‐6), 39.5 (C‐7), 72.8 (C‐9), and 28.9 (C‐12)], and three quaternary carbons [*δ*_C_: 201.5 (C‐2), 162.5 (C‐4), and 145.6 (C‐10)]. Collectively, these spectroscopic data indicated that compound **1** possessed a cadinane‐type sesquiterpene skeleton featuring an *α*,*β*‐unsaturated ketone moiety [12]. The enone unit was further supported by HMBC correlations (Figures 2 and S6) from H‐5 and H‐11 to C‐3/C‐4 and from H‐3 to C‐2. The ^1^H‐^1^H COSY spectrum revealed a spin system consisting of the H‐1/H‐6/H‐7/H‐8/H‐9 unit, with additional branching at H‐6 (to H‐5), H‐7 (to H‐12), and H‐12 (to H‐13 and H‐14) (Figures 2 and S7). An exocyclic methylene at the C‐10 position was established by the HMBC correlations from H‐15 to C‐1/C‐9/C‐10. The relative configuration of compound **1** was established from its coupling constant and NOESY data. The observed small coupling constant (*J* = 4.2 Hz) for H‐1 corresponds to the ^3^ *J* coupling between H‐1 and H‐6. Such a small vicinal coupling is consistent with an *α*‐orientation for both protons [13, 14]. In the NOESY spectrum (Figures 3 and S8), the correlations of H‐1 with H‐7 and H‐9 indicated that H‐7 and H‐9 also adopt an *α*‐orientation. The absolute configuration of **1** was assigned as 1*R*,6*R*,7*S*,9*S* by comparison of its experimental ECD spectrum with the calculated ECD curves (Figure 4). Accordingly, the structure of compound **1** was unambiguously assigned as shown in Figure 1 and designated viburetone A.

Compound **2** was obtained as a colorless oil. Its HRESIMS spectrum showed a sodium adduct ion at *m/z* 257.1521 [M + Na] ^+^ (calcd for C_15_H_22_O_2_Na^+^, 257.1512), establishing the molecular formula as C_15_H_22_O_2_ with five degrees of unsaturation (Figure S10). The IR spectrum (Figure S9) showed absorption bands for a hydroxyl group (3441 cm^−1^) and an *α*,*β*‐unsaturated carbonyl moiety (1660 cm^−1^). The ^1^H and ^13^C NMR spectra of **2** (Table 1 and Figures S11 and S12) closely resembled those of **1**, except for the absence of the oxygenated methine signal (H‐9/C‐9) and the appearance of an additional oxygenated quaternary carbon at *δ*_C_ 72.7 (C‐12). The planar structure was thus assigned as a cadinane‐type sesquiterpene bearing an *α*,*β*‐unsaturated ketone and an exocyclic methylene group. The enone moiety was confirmed by HMBC correlations (Figures 2 and S14) from H‐3 to C‐2 and from H‐5/H‐11 to C‐3/C‐4, while the exocyclic methylene was established by correlations from H‐15 to C‐1/C‐9/C‐10. All proton and carbon resonances were unambiguously assigned by combined analysis of HSQC, HMBC and ^1^H–^1^H COSY spectra (Figures S13–S15). The C‐12 resonance in **2** was shifted downfield by 43.8 ppm compared with that in **1**, consistent with hydroxylation at C‐12. This assignment was confirmed by HMBC correlations from H‐13 and H‐14 to C‐12. The relative configuration of compound **2** was supported by its small H‐1 coupling constant (*J*_1,6_ = 4.4 Hz) [13, 14] and the NOESY cross‐peak between H‐1 and H‐7 (Figures 3 and S16), which together indicated an *α*‐orientation for H‐1, H‐6, and H‐7. The absolute configuration was subsequently assigned as 1*R*,6*R*,7*R* by comparison of the experimental ECD spectrum with the calculated ECD curves (Figure 4). Therefore, compound **2** was identified as shown in Figure 1 and designated viburetone B.

**TABLE 1:** ^1^H and ^13^C NMR (600 and 150 MHz) data of compounds **1** and **2** in CDCl_3_.

No.	1		2	
	*δ*_H_, mult. (*J* in Hz)	*δ*_C_	*δ*_H_, mult. (*J* in Hz)	*δ*_C_
1	3.40, d (4.2)	49.8	2.87, d (4.4)	55.6
2		201.5		201.3
3	5.92, s	126.2	5.90, s	125.8
4		162.5		162.9
5	2.10, m (overlap) 1.94, dd (13.2, 4.2)	27.3	2.34, dd (13.5, 6.1) 2.26, dd (13.5, 4.3)	29.9
6	2.56, m	38.2	2.68, m	38.2
7	1.73, m	39.5	1.59, m	49.7
8	2.10, m (overlap) 1.47, m	33.6	1.86, m 1.59, m	23.7
9	4.38, t (3.0)	72.8	2.47, ddd (13.5, 4.3, 2.5) 2.10, ddd (13.5, 6.5, 3.7)	35.7
10		145.6		143.8
11	1.96, s	24.8	1.96, s	24.9
12	1.43, m	28.9		72.7
13	0.94, d (6.4)	20.9	1.28, s	30.2
14	0.95, d (6.4)	21.4	1.29, s	28.8
15	5.04, d (1.9) 4.52, d (1.9)	112.2	4.85, br s 4.41, br s	109.7

Fifteen known compounds were structurally identified as (3*S*,5*R*,6*S*,7*E*,9*R*)‐7‐megastigmene‐3,6,9‐triol (**3**) [15], (1*R*,2*S*,4*S*,6*R*)‐2,6‐dihydroxy‐1,8‐cineole (**4**) [16], wilsonol F (**5**) [17], viburnshosin A (**6**) [11], 28‐hydroxy‐*α*‐amyrin (**7**) [18], 28‐nor‐urs‐12‐ene‐3*β*,17*β*‐diol (**8**) [19], 3*β*‐hydroxyolean‐12‐en‐28‐oic acid (**9**) [20], (1*S*,2*R*)‐1‐(4‐hydroxy‐3‐methoxyphenyl)‐1‐ethoxypropan‐2‐ol (**10**) [21], (1*S*,2*S*)‐1‐(4‐hydroxy‐3‐methoxyphenyl)‐1‐ethoxypropan‐2‐ol (**11**) [21], (1*R*,2*R*)‐1‐(4‐hydroxy‐3‐methoxyphenyl)‐1‐methoxypropan‐2‐ol (**12**) [22], (7*R*,8*R*)‐threo‐1‐C‐syringylglycerol (**13**) [23], holophyllol B (**14**) [24], viburindrin D (**15**) [25], pinnatifidanin B V (**16**) [26], and (+)‐7′‐methoxylariciresinol (**17**) [27], by matching their spectroscopic and physical characteristics with literature values.

**FIGURE 5:**
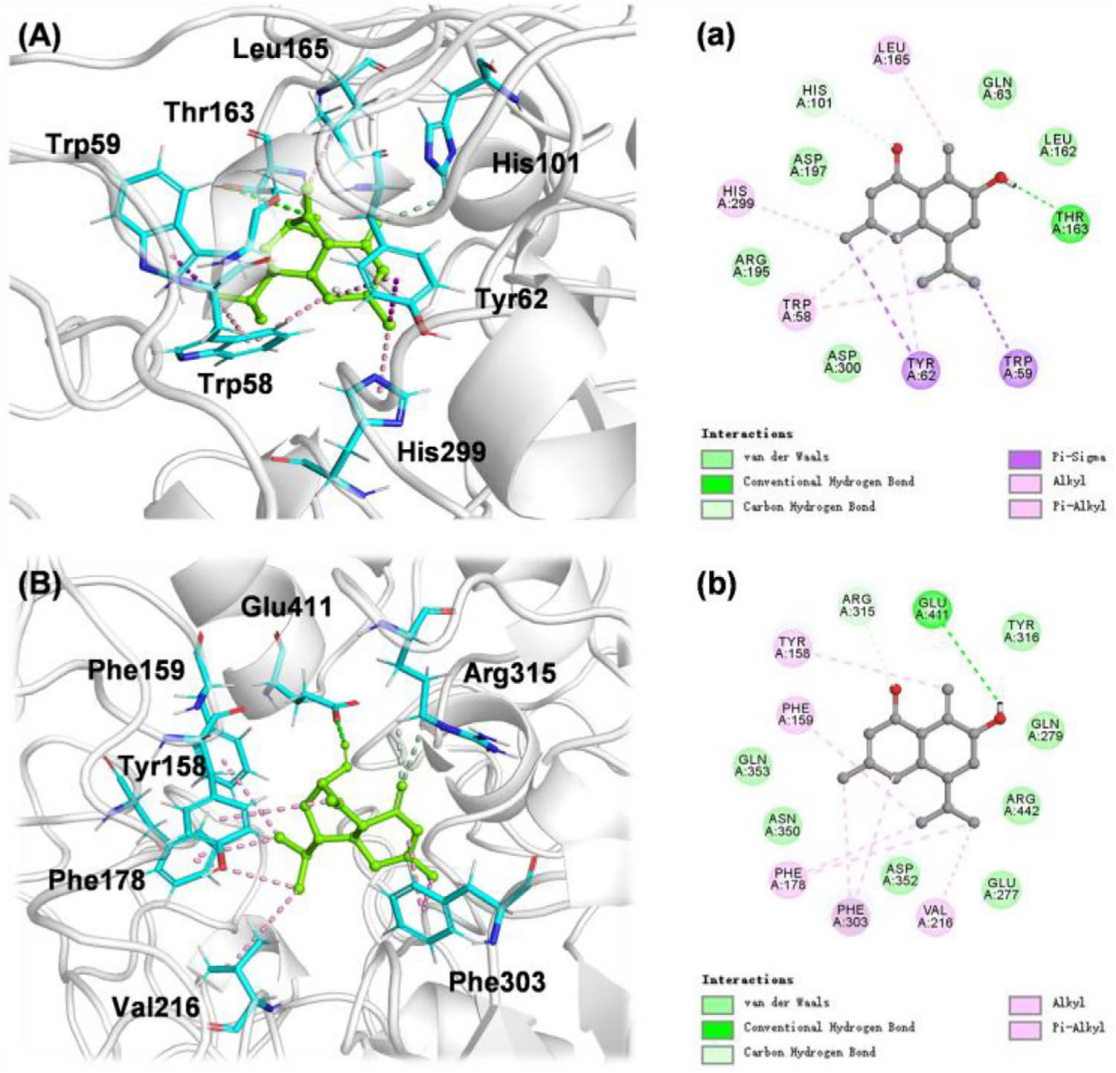
Molecular docking of compound **1** with *α*‐amylase (A, a) and *α*‐glucosidase (B, b). (A) Three‐dimensional binding pose of compound **1** in the active site of *α*‐amylase; (a) Two‐dimensional diagram of key interactions between compound **1** and *α*‐amylase residues. (B) Three‐dimensional binding pose of compound **1** in the active site of *α*‐glucosidase; (b) Two‐dimensional diagram of key interactions between compound **1** and *α*‐glucosidase residues.

The isolated compounds (**1**–**17**) were tested for their ability to inhibit *α*‐amylase and *α*‐glucosidase (Table 2). Among them, compounds **7** and **8** inhibited *α*‐amylase with IC_50_ values of 21.67 and 19.65 µM, respectively, exhibiting greater potency than acarbose (IC_50_ = 39.19 µM), while compound **1** (IC_50_ = 38.20 µM) showed comparable activity. Against *α*‐glucosidase, compound **14** (IC_50_ = 20.37 µM) was considerably more potent than acarbose (IC_50_ = 25.73 µM), whereas compound **1** (IC_50_ = 24.15 µM) displayed inhibitory activity comparable to that of the positive control. Interestingly, compound **1** exhibited dual inhibitory activity against both enzymes, and it was more potent than compound **2** (*α*‐amylase: IC_50_ = 45.16 µM; *α*‐glucosidase: IC_50_ = 36.41 µM), which may be attributed to the position of the hydroxyl group, which is located at C‐9 in compound **1** but at C‐12 in compound **2**. Compounds **7** and **8** showed stronger *α*‐amylase inhibitory activity than compound **9** (IC_50_ = 85.16 µM), likely due to differences in the positions of the methyl groups on ring E of the pentacyclic triterpenoid scaffold: compounds **7** and **8** belong to the ursane‐type triterpenes, whereas compound **9** is oleanane‐type. Additionally, compound **14** displayed stronger *α*‐glucosidase inhibitory activity than compound **15** (IC_50_ = 29.46 µM), which may be attributed to the presence of an additional hydroxyl group at C‐7′ in its planar structure and/or the different configuration at C‐8′ in its stereostructure. Molecular docking analysis demonstrated that compound **1** stably occupies the active sites of *α*‐amylase and *α*‐glucosidase (Figure 5). The *α*‐amylase‐**1** complex was stabilized mainly by hydrogen bonds with the residue Thr163 as well as multiple hydrophobic interactions including Pi‐Sigma interactions with Trp59 and Tyr62, Alkyl/Pi‐Alkyl interactions with Trp58, His101, Leu165, and His299. In addition, compound **1** was stably bound to *α*‐glucosidase through strong hydrogen bonds with the key catalytic residue Glu411, hydrophobic forces with Tyr158, Phe159, Phe178, Val216, Phe303, Arg315, and additional van der Waals interactions that contribute to stabilization.

**TABLE 2:** The *α*‐amylase and *α*‐glucosidase inhibitory activities of compounds **1**‒**17**.

Comp.	IC_50_ (µM)		Comp.	IC_50_ (µM)	
	*α‐*amylase	*α‐*glucosidase		*α‐*amylase	*α‐*glucosidase
**1**	38.20 ± 1.16^f^	24.15 ± 1.12^e^	**10**	>100	51.38 ± 1.54^a^
**2**	45.16 ± 1.34^e^	36.41 ± 0.91^c^	**11**	>100	53.52 ± 2.03^a^
**3**	>100	>100	**12**	>100	50.11 ± 1.67^ab^
**4**	>100	>100	**13**	>100	48.63 ± 1.48^b^
**5**	>100	>100	**14**	>100	20.37 ± 0.83^f^
**6**	>100	>100	**15**	>100	29.46 ± 0.69^d^
**7**	21.67 ± 1.03^g^	>100	**16**	70.42 ± 2.75^c^	35.18 ± 1.24^c^
**8**	19.65 ± 0.91^g^	>100	**17**	60.54 ± 2.39^d^	46.93 ± 1.79^b^
**9**	85.16 ± 2.27^a^	>100	Acarbose	39.19 ± 1.28^f^	25.73 ± 0.85^e^

IC_50_ values are presented as mean ± SD (*n* = 3). Different superscript letters (a–g) in the same column indicate statistically significant differences at *p* < 0.05 (one‑way ANOVA followed by Duncan's multiple range test). Acarbose was used as the positive control for both enzyme assays.

## 3 Conclusions

This work presents the first detailed phytochemical analysis of VMM flowers, leading to the characterization of seventeen compounds, including two new cadinane‐type sesquiterpenes, viburetones A (**1**) and B (**2**). Compounds **3**–**5**, **10**–**14**, **16**, and **17** were isolated from the family Adoxaceae for the first time. In the biological evaluation, compound **1** exhibited dual inhibitory activity against *α*‐amylase and *α*‐glucosidase comparable to that of the positive control acarbose. Molecular docking suggested that compound **1** stably bound to the active sites of both enzymes through hydrogen bonding and hydrophobic interactions. These findings expand the chemical diversity of the genus *Viburnum*, in part by reporting the first cadinane‐type sesquiterpenes from this genus, and identify VMM flowers as a promising source of bioactive constituents for further investigation as *α*‐amylase and *α*‐glucosidase inhibitors.

## 4 Experimental Section

### 4.1 General experimental procedures

Optical rotation and circular dichroism (CD) measurements were performed on a JASCO P‐1020 digital polarimeter and a J‐810 spectropolarimeter (Tokyo, Japan), respectively. NMR experiments were conducted on a 600 MHz Bruker Avance spectrometer (Bruker, Rheinstetten, Germany). High‐resolution electrospray ionization mass spectrometry (HRESIMS) data were acquired using a Bruker MaXis QTOF apparatus (Bruker, Bremen, Germany). Analytical HPLC was carried out on an Agilent 1100/1260 system fitted with a YMC‐Pack ODS‐A column (250 × 4.6 mm, 5 µm), and semipreparative HPLC was performed on an LC3000 system (Chuang Xin Tong Heng Co., Beijing, China) equipped with a YMC ODS‐AQ column (250 × 20 mm, 5 µm). All biochemical reagents were obtained from Sigma‐Aldrich (St. Louis, MO, USA).

### 4.2 Plant material

The fresh flowers of *V. macrocephalum* f. *macrocephalum* were collected in Yangzhou, China, in May 2024 and authenticated by Professor M. X. Liu from Yangzhou University. A voucher specimen (accession number 20240502) has been deposited in the authors’ laboratory at Yangzhou University for future reference.

### 4.3 Extraction and isolation

The air‐dried flowers of VMM (1.1 kg) were extracted with MeOH (3 × 10 L) at room temperature, each time for 24 h. After filtration, the combined extract was concentrated under reduced pressure to give 105 g of crude extract. The crude extract was then subjected to silica gel column chromatography, eluting with a gradient of CH_2_Cl_2_–MeOH (50:1→1:1, v/v) to afford seven fractions (Fr. 1–Fr. 7). Fraction 2 (15.1 g) was separated by silica gel CC using a gradient of PE–EtOAc (30:1→1:1, v/v), affording subfractions Fr. 2‐1–Fr. 2‐7. Fr. 2‐2 and Fr. 2‐4 were each purified on a Sephadex LH‐20 column with CH_2_Cl_2_–MeOH (2:1, v/v) as the eluent, yielding compounds **7** (4 mg) and **8** (5 mg) from Fr. 2‐2, and compound **9** (239 mg) from Fr. 2‐4, respectively. Fraction 3 (29.7 g) was also subjected to silica gel CC, eluted with PE–EtOAc (50:1→1:1, v/v), to give subfractions Fr. 3‐1–Fr. 3‐8. Fr. 3‐2 was further separated on Sephadex LH‐20 (CH_2_Cl_2_–MeOH, 2:1, v/v) into two subfractions (Fr. 3‐2‐1 and Fr. 3‐2‐2). Fr. 3‐2‐1 was then fractionated by reversed‐phase (ODS) CC using a MeOH–H_2_O gradient (40:60→90:10, v/v), producing six subfractions (Fr. 3‐2‐1‐1–Fr. 3‐2‐1‐6). Among these, Fr. 3‐2‐1‐1, Fr. 3‐2‐1‐2, Fr. 3‐2‐1‐3, and Fr. 3‐2‐1‐4 were each purified by repeated Sephadex LH‐20 (CH_2_Cl_2_–MeOH, 2:1, v/v) to afford compound **3** (21 mg); compounds **5** (37 mg) and **6** (45 mg); compound **4** (11 mg); and compounds **1** (35 mg) and **2** (8 mg), respectively. Similarly, Fr. 3‐2‐2 was separated by ODS CC (MeOH–H_2_O, 40:60→90:10, v/v) into four subfractions (Fr. 3‐2‐2‐1→Fr. 3‐2‐2‐4). Fr. 3‐2‐2‐2 was purified on Sephadex LH‐20 (CH_2_Cl_2_–MeOH, 2:1, v/v) to afford compounds **10** (28 mg) and **11** (5 mg), while Fr. 3‐2‐2‐3 was similarly purified to yield compound **12** (9 mg). Fr. 3‐4 was purified by ODS CC with a MeOH–H_2_O gradient (20:80→90:10, v/v), giving six subfractions (Fr. 3‐4‐1→Fr. 3‐4‐6). Fr. 3‐4‐2 was processed on Sephadex LH‐20 (CH_2_Cl_2_–MeOH, 2:1, v/v) to provide compounds **13** (6 mg) and **16** (13 mg). Fr. 3‐4‐3 underwent the same Sephadex LH‐20 treatment followed by preparative HPLC (MeOH–H_2_O, 47:53, v/v) to give **14** (15 mg, t*_R_* = 31.8 min), and another preparative HPLC (MeOH–H_2_O, 40:60, v/v) to yield **17** (7 mg, t*_R_* = 34.9 min). Finally, Fr. 3‐4‐4 was purified on Sephadex LH‐20 under identical conditions to yield compound **15** (12 mg).

Viburetone A (**1**): Colorless oil; ${\left[\alpha \right]}_{D}^{23}$ = −18.1 (*c* 0.15, MeOH); HRESIMS *m/z* 257.1532 [M+Na]^+^ (calcd for C_15_H_22_O_2_Na^+^, 257.1512); UV (MeOH) *λ*_max_ (log *ε*) 236 (2.98) nm; CD (MeOH) *λ* (Δ*ε*) 202 (3.68), 234 (3.40), 329 (−0.16) nm; IR (KBr) *v*_max_ 3407, 3078, 2958, 2921, 1665, 1435, 1383, 1305, 1275, 1035, 905, 550 cm^−1^; ^1^H NMR and ^13^C NMR data, see Table 1.

Viburetone B (**2**): Colorless oil; ${\left[\alpha \right]}_{D}^{23}$ = −12.6 (*c* 0.10, MeOH); HRESIMS *m/z* 257.1521 [M+Na]^+^ (calcd for C_15_H_22_O_2_Na^+^, 257.1512); UV (MeOH) *λ*_max_ (log *ε*) 236 (3.04) nm; CD (MeOH) *λ* (Δ*ε*) 213 (−0.51), 236 (4.52), 271 (−0.41), 327 (−0.33) nm; IR (KBr) *v*_max_ 3441, 3078, 2972, 2940, 1660, 1438, 1381, 1301, 1266, 1176, 892, 547 cm^−1^; ^1^H NMR and ^13^C NMR data, see Table 1.

### 4.4 ECD calculation

A systematic conformational search of compounds **1** and **2** was performed [5], and conformers consistent with the relative configuration inferred from NMR data were selected. These conformers were geometrically optimized and subjected to frequency analysis at the density functional theory (DFT) level (B3LYP/6‐31G(d,p)), followed by Boltzmann weighting based on their free energies after confirming the absence of imaginary frequencies. Subsequently, TDDFT calculations (at the CAM‐B3LYP/6‐311G(d,p) level and including solvent effects via CPCM in MeOH) were performed on the optimized structures. Finally, the theoretical ECD spectrum was obtained using the SpecDis 1.71 program by weighting and summing the simulated spectra of all conformers.

### 4.5 Carbohydrate hydrolase inhibitory assay

The inhibitory activities of the isolated compounds against *α*‐amylase (porcine pancreas) and *α*‐glucosidase (rat intestine) were evaluated by standard colorimetric assays [5]. To evaluate *α*‐amylase inhibition, test compounds (5–100 µM) were pre‐incubated with *α*‐amylase (2 U/mL) in 20 mM sodium phosphate buffer (pH 6.9) at 37 °C for 10 min. The enzymatic reaction was initiated by addition of 2% (w/v) soluble starch and allowed to proceed for 15 min. It was then terminated by adding 3,5‐dinitrosalicylic acid (DNS) reagent, followed by heating at 100 °C for 15 min. Absorbance was measured at 540 nm. In the *α*‐glucosidase inhibition assay, compounds were pre‐incubated with *α*‐glucosidase (1 U/mL) under the same conditions, followed by the addition of 5 mM p‐nitrophenyl‐*α*‐d‐glucopyranoside (*p*‐NPG) as substrate. After 30 min of incubation at 37 °C, the reaction was quenched with an equal volume of 1 M Na_2_CO_3_, and the absorbance of the released *p*‐nitrophenol was recorded at 405 nm. Acarbose was employed as a positive control in both assays.

### 4.6 Molecular docking study

The three‐dimensional structures of *α*‐amylase (PDB ID: 4W93) and *α*‐glucosidase (PDB ID: 3A4A) were retrieved from the Protein Data Bank (PDB). Prior to docking, all co‐crystallized ligands, ions, and water molecules were removed, and polar hydrogen atoms were added. The 3D conformations of the test compounds were energy‐minimized using Chem3D Ultra 20.0 (PerkinElmer). Molecular docking simulations were carried out with AutoDock Vina [5], employing a grid box centered on the catalytic active site. The top‐scoring poses were selected based on binding affinity (kcal/mol) and consistency of key intermolecular interactions. Protein–ligand complexes were visualized and analyzed using PyMOL (Schrödinger) and BIOVIA Discovery Studio 2019.

### 4.7 Statistical analysis

All experimental data were expressed as mean ± standard deviation (SD) (*n* = 3). Statistical analysis was performed using SPSS 25.0 software. Differences between groups were assessed by one‐way ANOVA, and multiple comparisons were performed using Duncan's multiple range test. A *p*‐value < 0.05 was considered statistically significant.

## Author Contributions

**Ya‐Jiao Chen**: investigation, methodology. **Hong‐Juan Zhou**: formal analysis, validation. **Xue‐Jing Zhu**: software, visualization. **Jia Chen**: methodology, validation, writing – original draft. **Jian‐Hua Shao**: funding acquisition, supervision. **Chun‐Chao Zhao**: project administration, funding acquisition, writing – review and editing.

## Conflicts of Interest

The authors declare no conflicts of interest.

## Supporting information




**Supporting File 1**: cbdv71650‐sup‐0001‐SuppMat.pdf

## Data Availability

The data that support the findings of this study are available in the Supporting Information of this article.
