# Supplementary material for: Constituents From the Flowers of Viburnum macrocephalum f. macrocephalum and Their α‐Amylase and α‐Glucosidase Inhibitory Activities
Source: Chem Biodivers. 2026 Aug 31;23(9):e71650. doi: 10.1002/cbdv.71650 (PMC13529240; doi:10.1002/cbdv.71650)
Supplement: Supplementary file 1 [file CBDV-23-e71650-s001.pdf]

SUPPLEMENTARY MATERIAL

Constituents from the Flowers of *Viburnum macrocephalum* f. *macrocephalum* and their *α*-Amylase and *α*-Glucosidase Inhibitory Activities

Ya-Jiao Chen[+], Hong-Juan Zhou[+], Xue-Jing Zhu, Jia Chen*, Jian-Hua Shao, and Chun-Chao Zhao*

College of Bioscience and Biotechnology, Yangzhou University, Yangzhou 225009, China

*Corresponding Authors. **Jia Chen:** College of Bioscience and Biotechnology, Yangzhou University, Yangzhou 225009, China; e-mail: jchen92@yzu.edu.cn (J. Chen); **Chun-Chao Zhao:** College of Bioscience and Biotechnology, Yangzhou University, Yangzhou 225009, China; e-mail: cczhao@yzu.edu.cn (C. Zhao). [+] These authors contributed equally to this work.

ABSTRACT

Phytochemical investigation of the flowers of *Viburnum macrocephalum* f. *macrocephalum* (VMM), a sterile horticultural form native to China, led to the isolation of two new cadinane-type sesquiterpenes, viburetones A (**1**) and B (**2**), which represent the first examples of this skeletal type from the genus *Viburnum*, together with fifteen known compounds (**3**–**17**). The structures of the new compounds were elucidated by comprehensive analysis of their 1D and 2D NMR, HRESIMS, and ECD data. All isolates were evaluated for their inhibitory activities against *α*-amylase and *α*-glucosidase, key enzymes involved in carbohydrate metabolism. Among them, the new compound **1** exhibited inhibitory activities against *α*-amylase (IC_{50} = 38.20 μM) and *α*-glucosidase (IC_{50} = 24.15 μM), comparable to those of acarbose (IC_{50} = 39.19 and 25.73 μM, respectively). Molecular docking studies suggested that compound **1** bound to the active sites of both enzymes via hydrogen bonding and hydrophobic interactions.

KEYWORDS: *Viburnum macrocephalum* f. *macrocephalum*; phytochemical constituents; *α*-amylase inhibition; *α*-glucosidase inhibition

List of supporting information

**1.**	**Figure S1**. The IR Spectrum of Compound **1**
**2**.	**Figure S2**. The (+)-HRESIMS Spectrum of Compound **1**
**3**.	**Figure S3**. The ^{1}H NMR Spectrum of Compound **1** in $CDCl_3$
**4**.	**Figure S4**. The ^{13}C NMR Spectrum of Compound **1** in $CDCl_3$
**5**.	**Figure S5**. The HSQC Spectrum of Compound **1** in $CDCl_3$
**6**.	**Figure S6**. The HMBC Spectrum of Compound **1** in $CDCl_3$
**7**.	**Figure S7**. The ^{1}H-^{1}H COSY Spectrum of Compound **1** in $CDCl_3$
**8**.	**Figure S8**. The NOESY Spectrum of Compound **1** in $CDCl_3$
**9**.	**Figure S9**. The IR Spectrum of Compound **2**
**10**.	**Figure S10**. The (+)-HRESIMS Spectrum of Compound **2**
**11**.	**Figure S11**. The ^{1}H NMR Spectrum of Compound **2** in $CDCl_3$
**12**.	**Figure S12**. The ^{13}C NMR Spectrum of Compound **2** in $CDCl_3$
**13**.	**Figure S13**. The HSQC Spectrum of Compound **2** in $CDCl_3$
**14**.	**Figure S14**. The HMBC Spectrum of Compound **2** in $CDCl_3$
**15**.	**Figure S15**. The ^{1}H-^{1}H COSY Spectrum of Compound **2** in $CDCl_3$
**16**.	**Figure S16**. The NOESY Spectrum of Compound **2** in $CDCl_3$

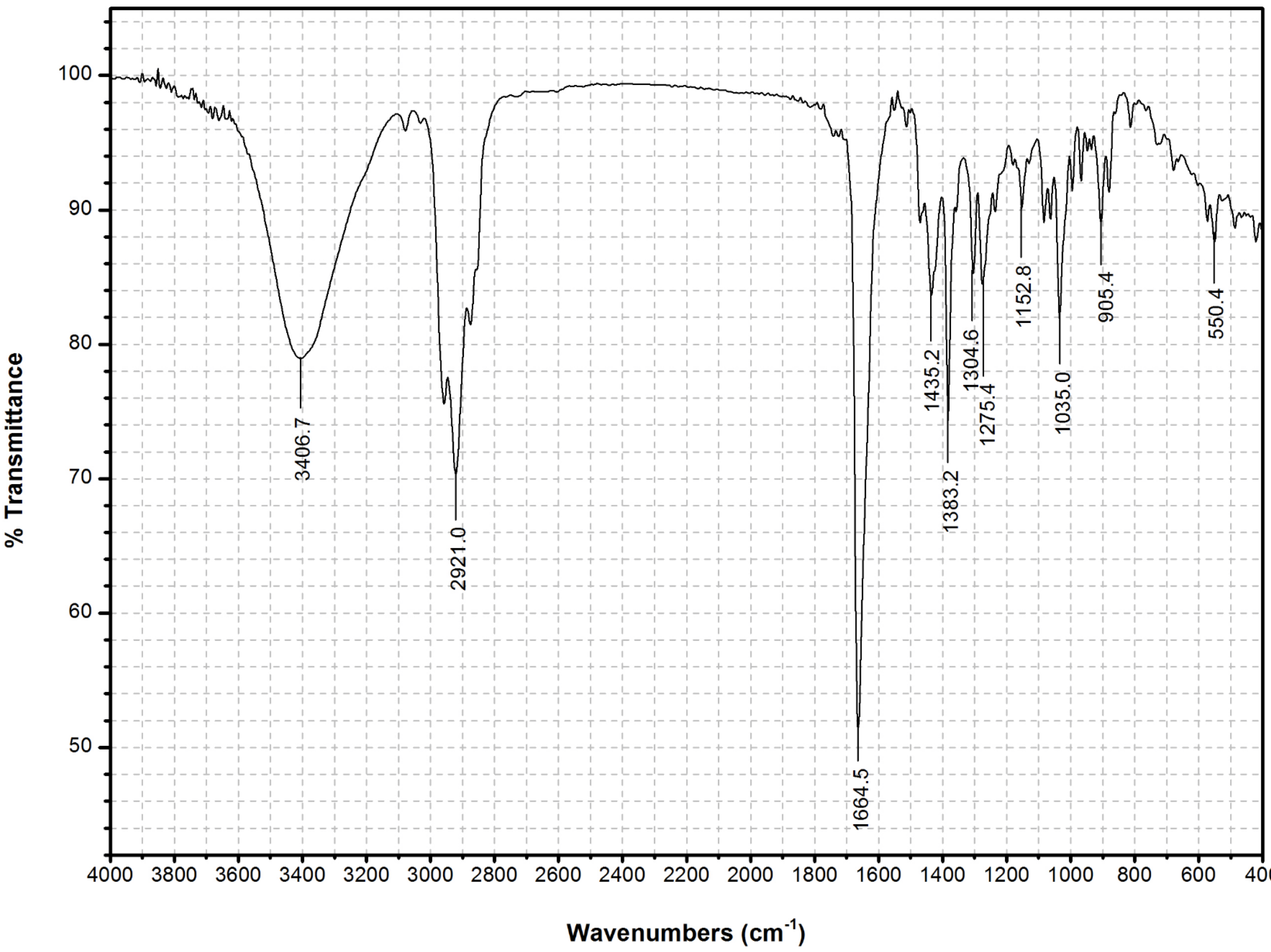

Figure S1. The IR Spectrum of Compound **1**

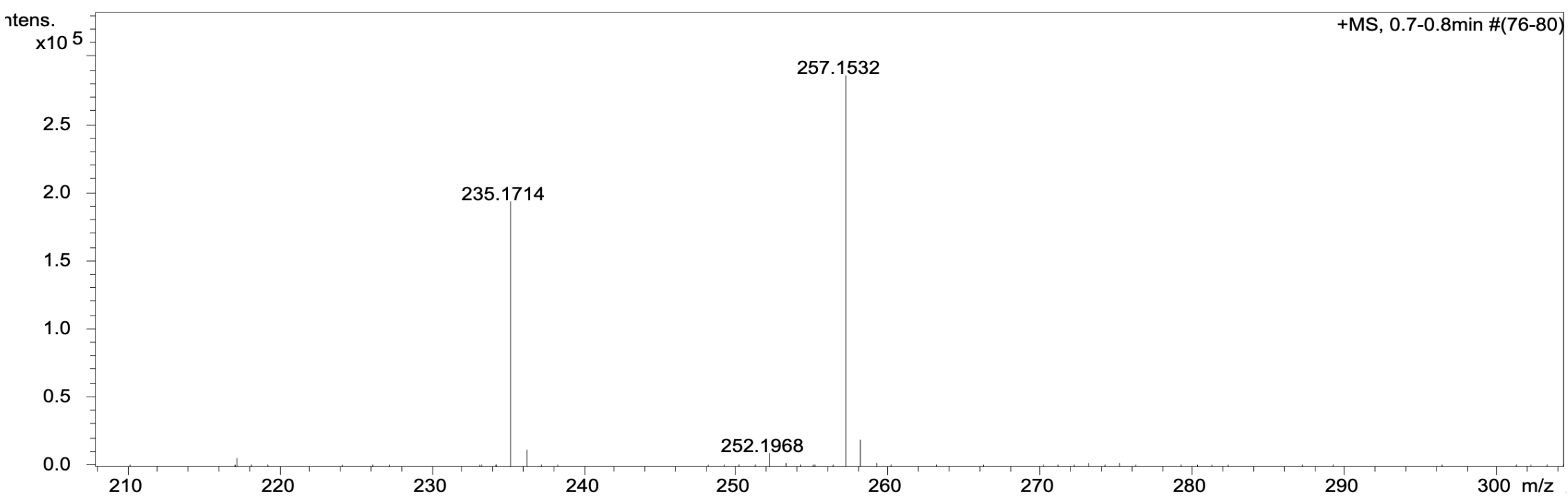

Meas. m/z	#	Formula	Score	m/z	err [mDa]	err [ppm]	mSigma	rdb	e⁻ Conf	N-Rule
257.1532	1	C 15 H 22 Na O 2	100	257.1512	-2	-7.6	56.6	4.5	even	ok

Figure S2. The (+)-HRESIMS Spectrum of Compound **1**

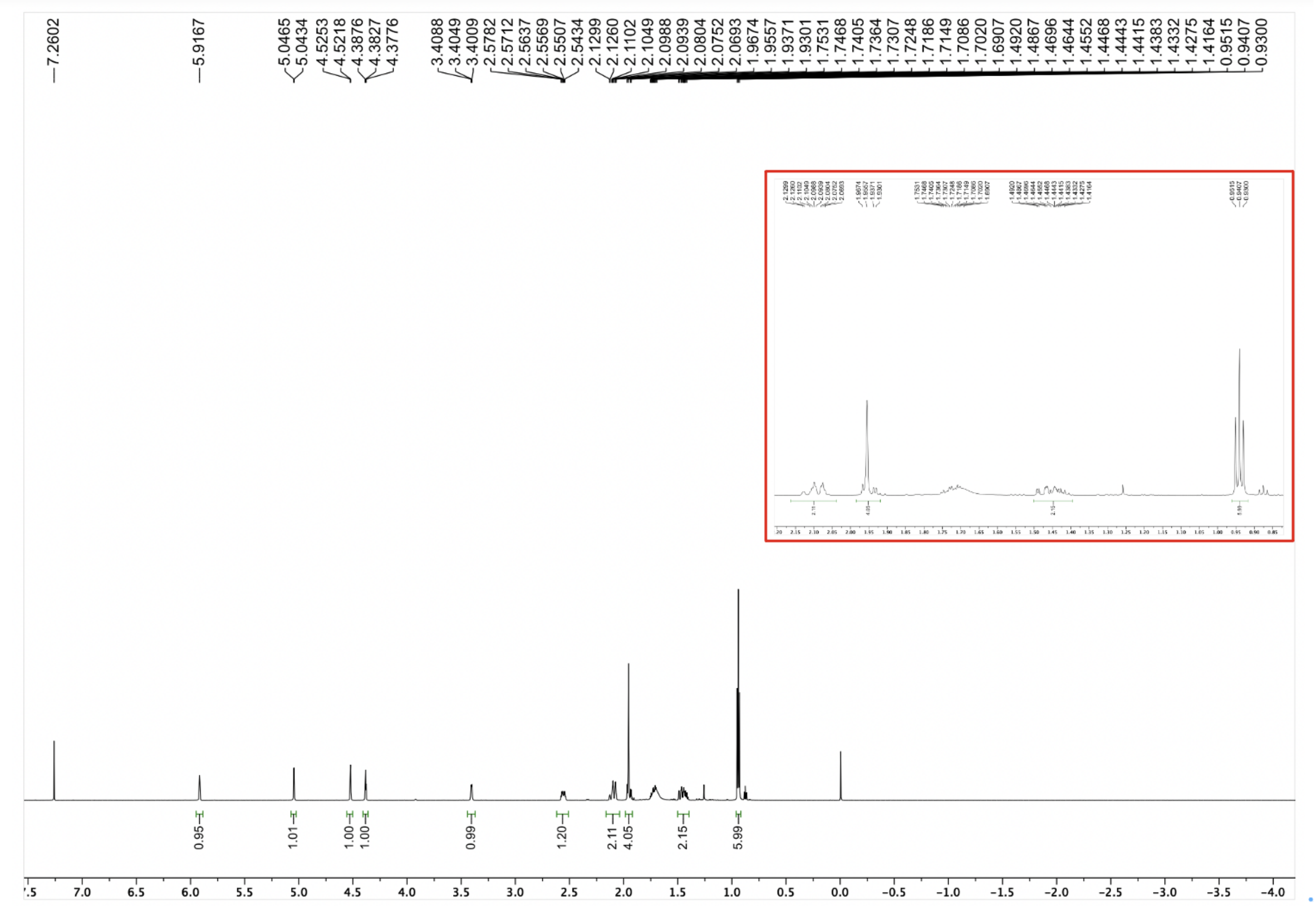

Figure S3. The ^{1}H NMR Spectrum of Compound **1** in $CDCl_3$

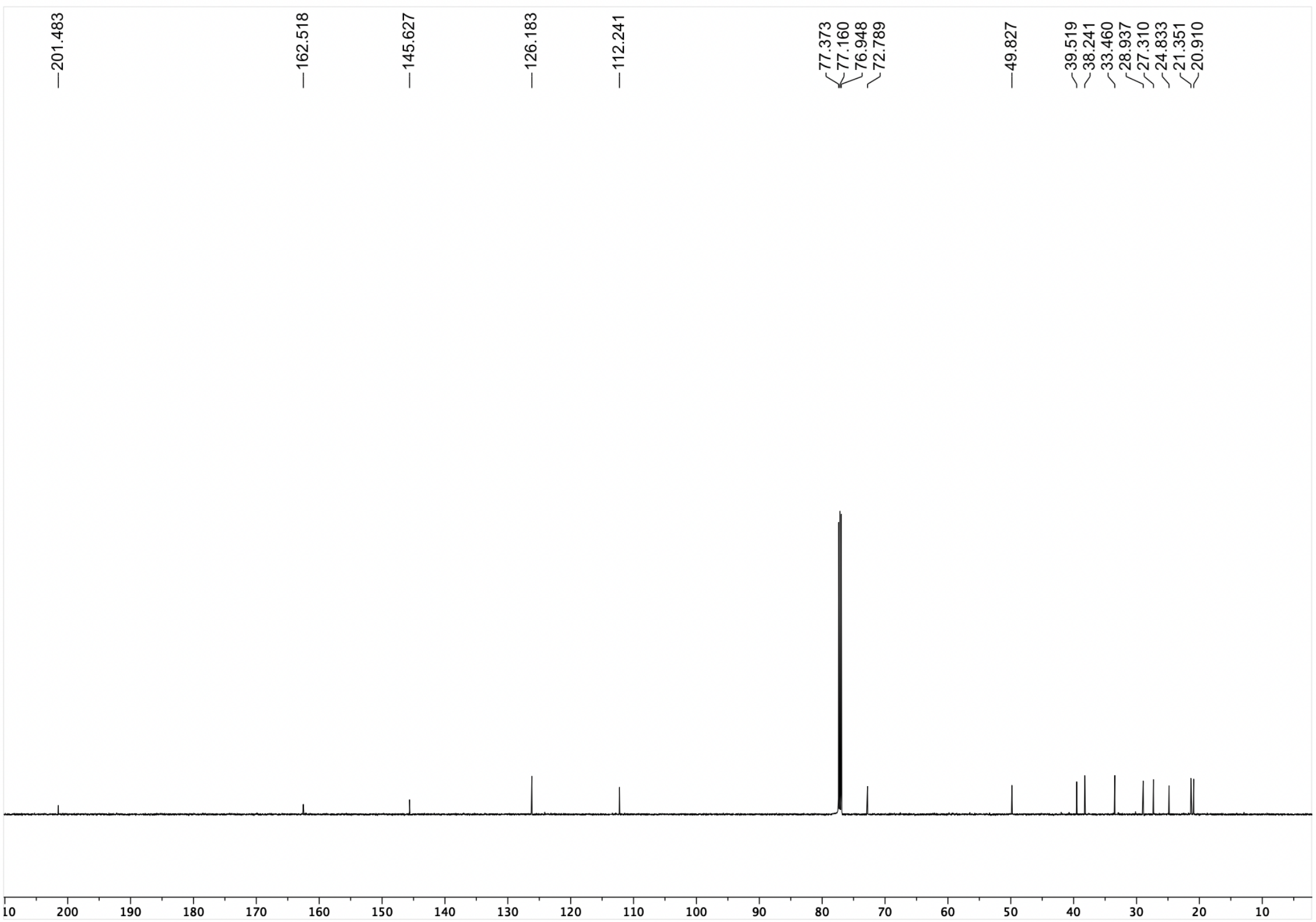

Figure S4. The ^{13}C NMR Spectrum of Compound **1** in $CDCl_3$

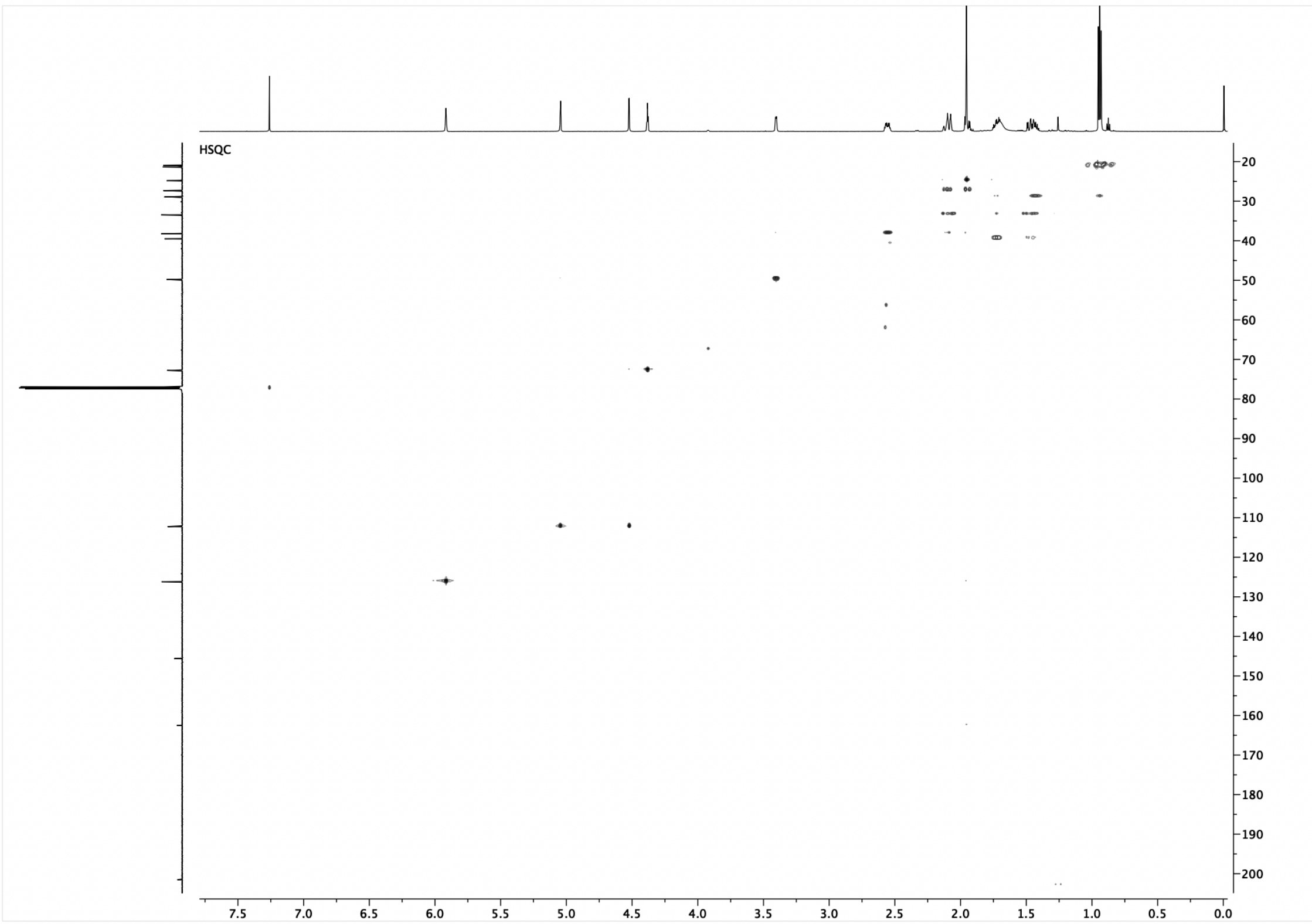

Figure S5. The HSQC Spectrum of Compound **1** in $CDCl_3$

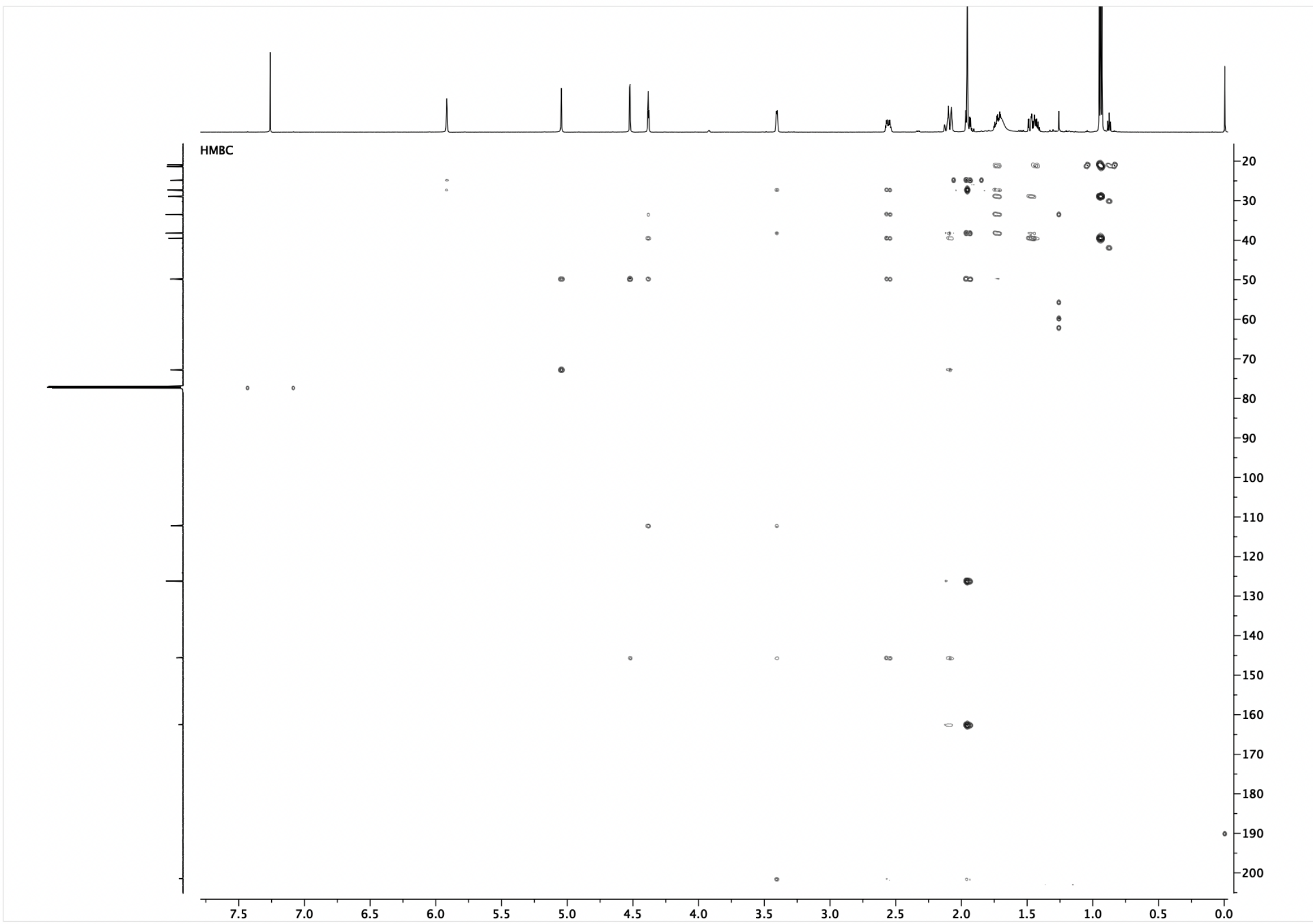

Figure S6. The HMBC Spectrum of Compound **1** in $CDCl_3$

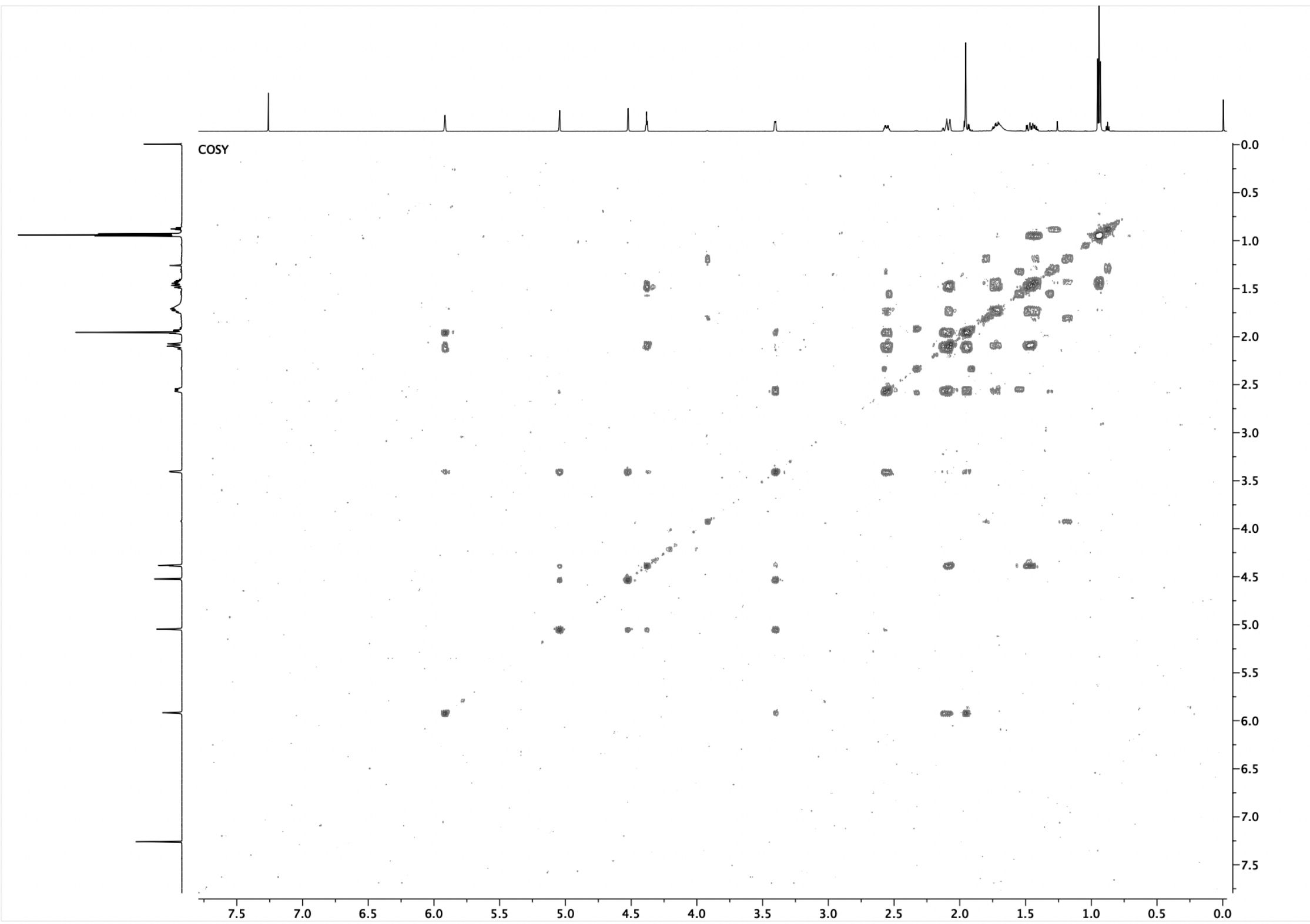

Figure S7. The ^{1}H-^{1}H COSY Spectrum of Compound **1** in $CDCl_3$

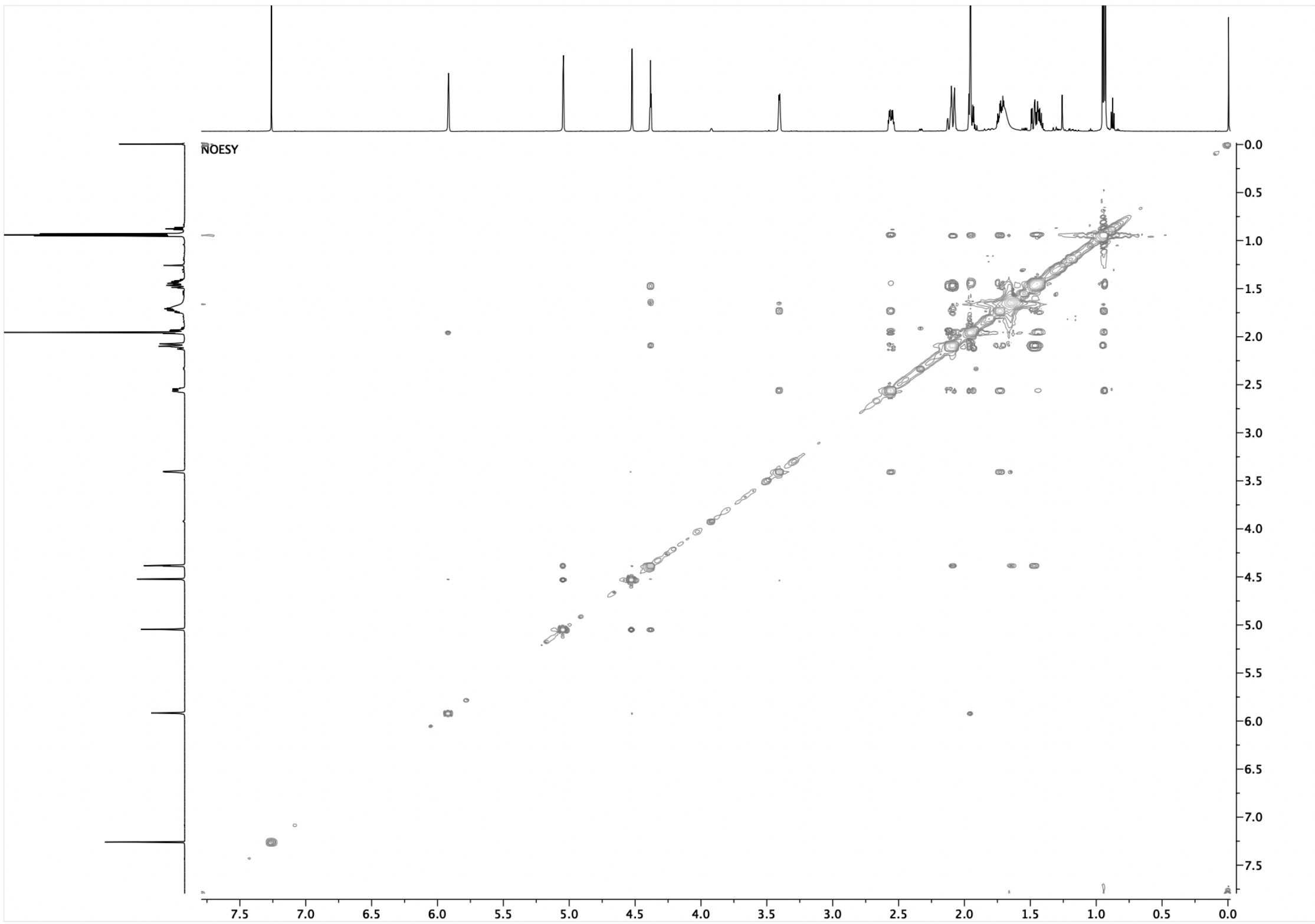

Figure S8. The NOESY Spectrum of Compound **1** in $CDCl_3$

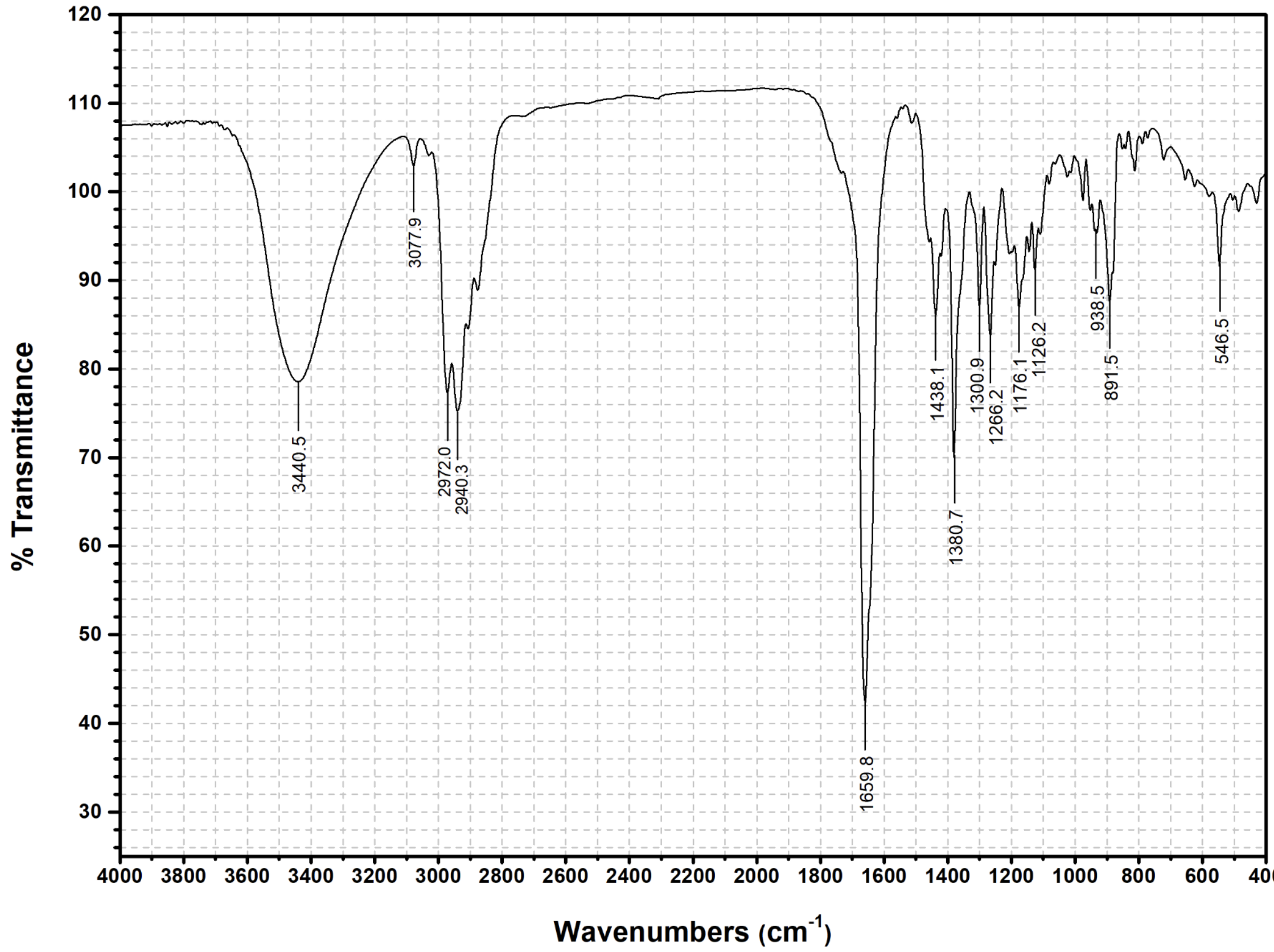

Figure S9. The IR Spectrum of Compound **2**

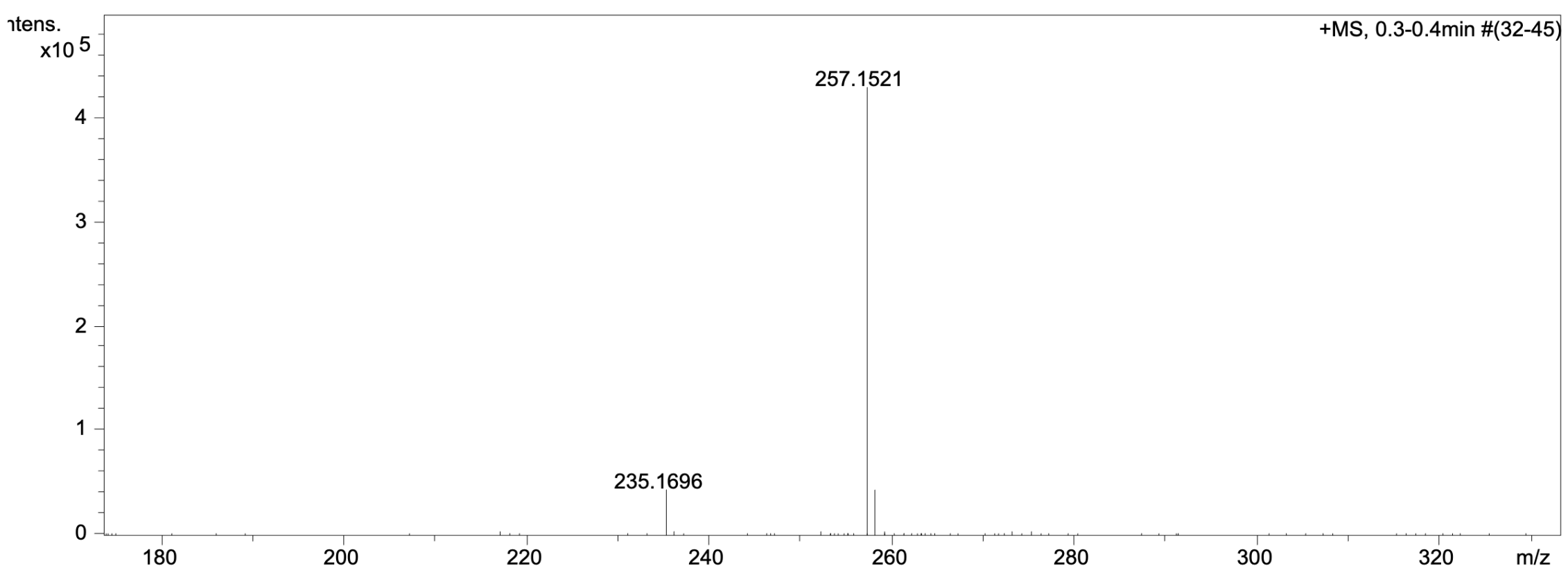

Meas. m/z	#	Formula	Score	m/z	err [mDa]	err [ppm]	mSigma	rdb	e⁻ Conf	N-Rule
257.1521	1	C 15 H 22 Na O 2	100	257.1512	-0.9	-3.4	38.6	4.5	even	ok

Figure S10. The (+)-HRESIMS Spectrum of Compound **2**

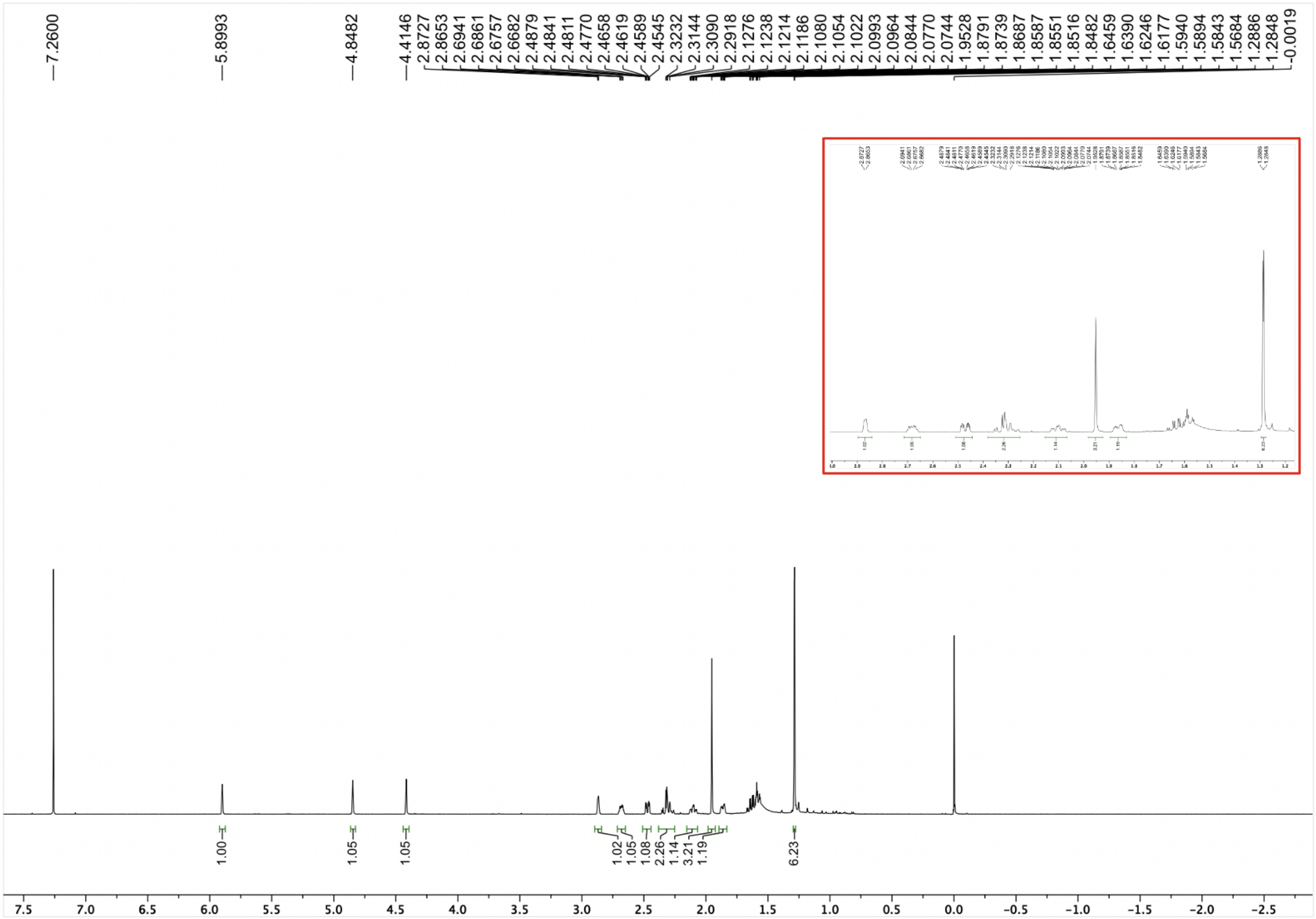

Figure S11. The ^{1}H NMR Spectrum of Compound **2** in $CDCl_3$

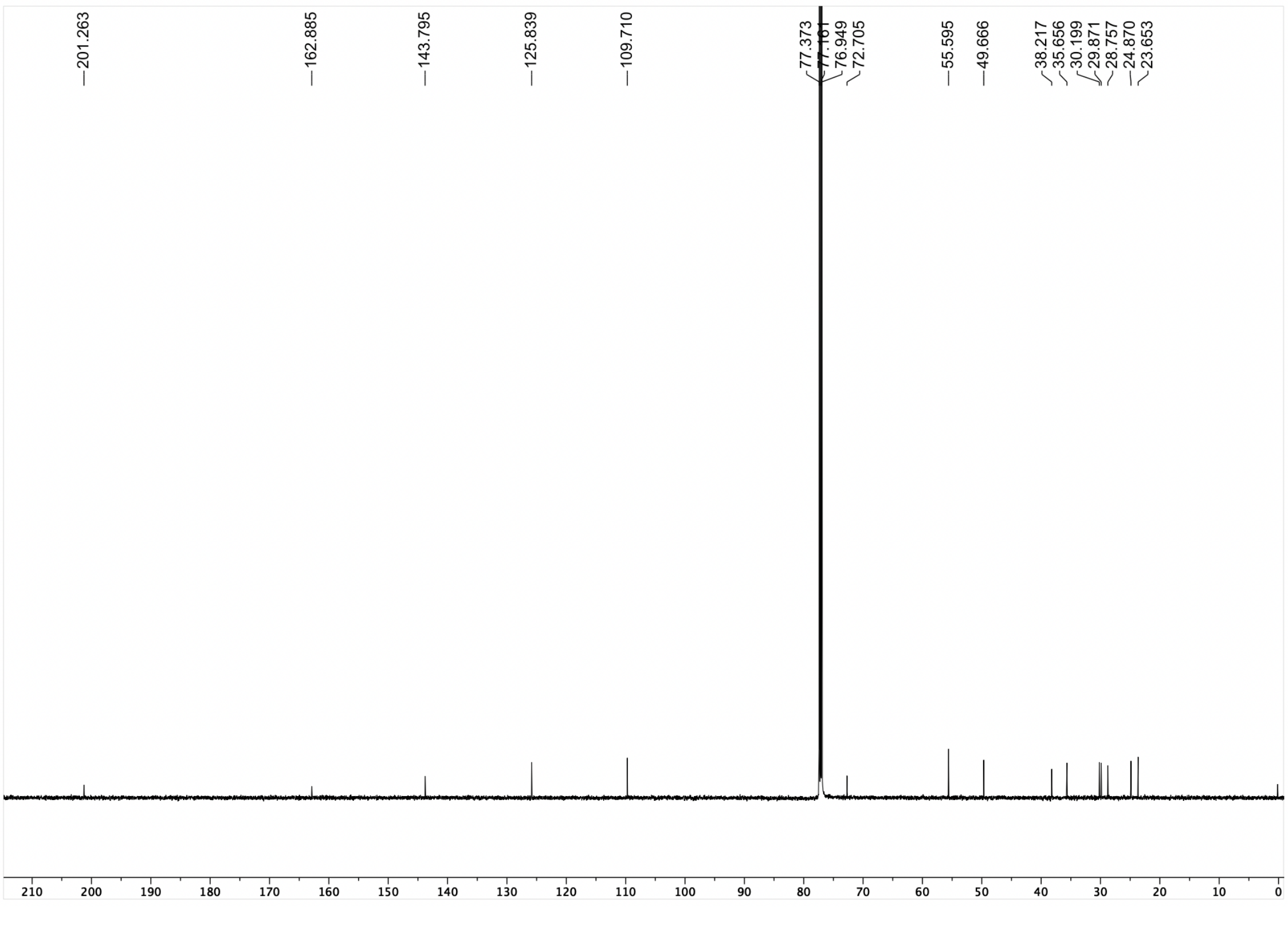

Figure S12. The ^{13}C NMR Spectrum of Compound **2** in $CDCl_3$

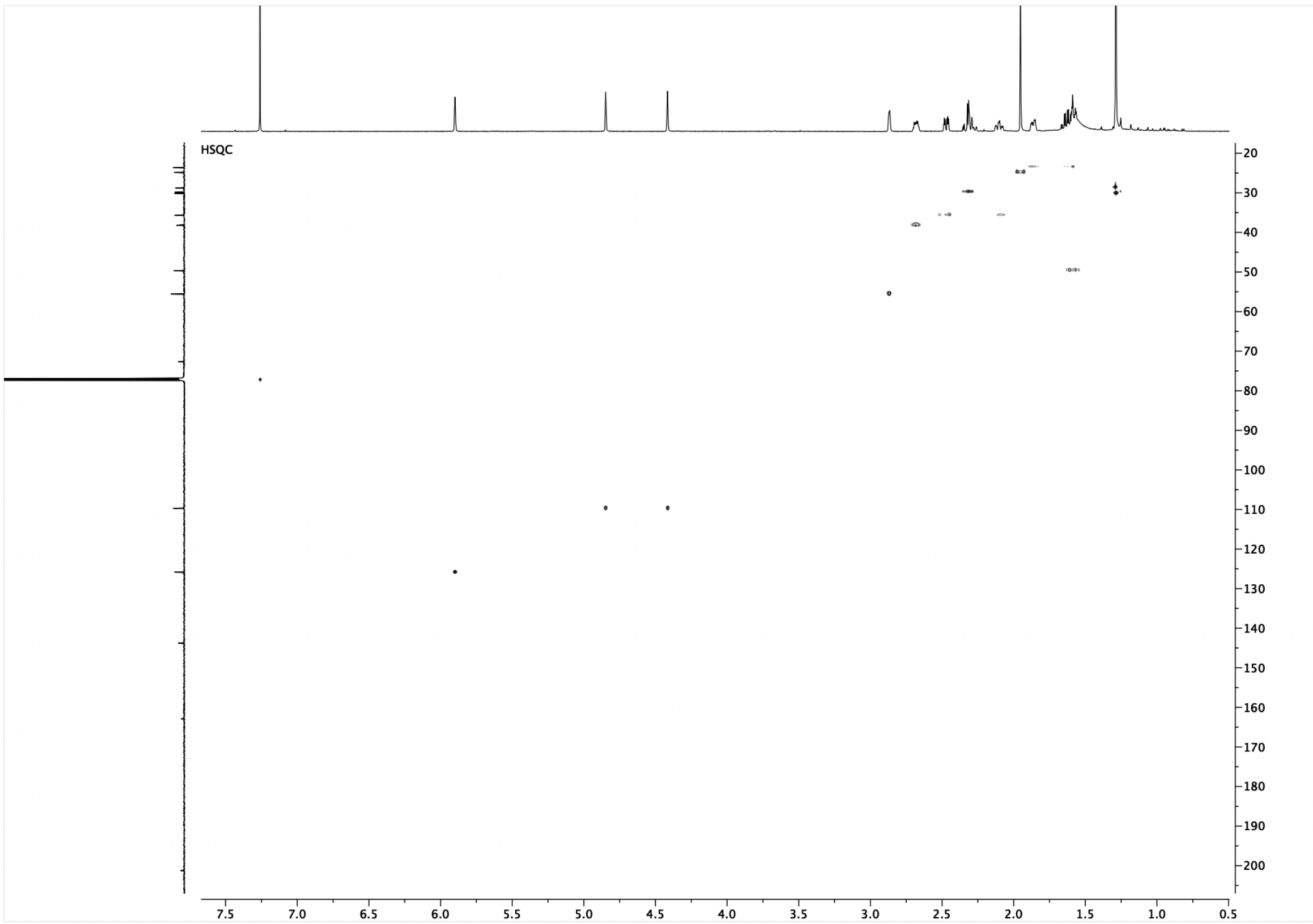

Figure S13. The HSQC Spectrum of Compound **2** in $CDCl_3$

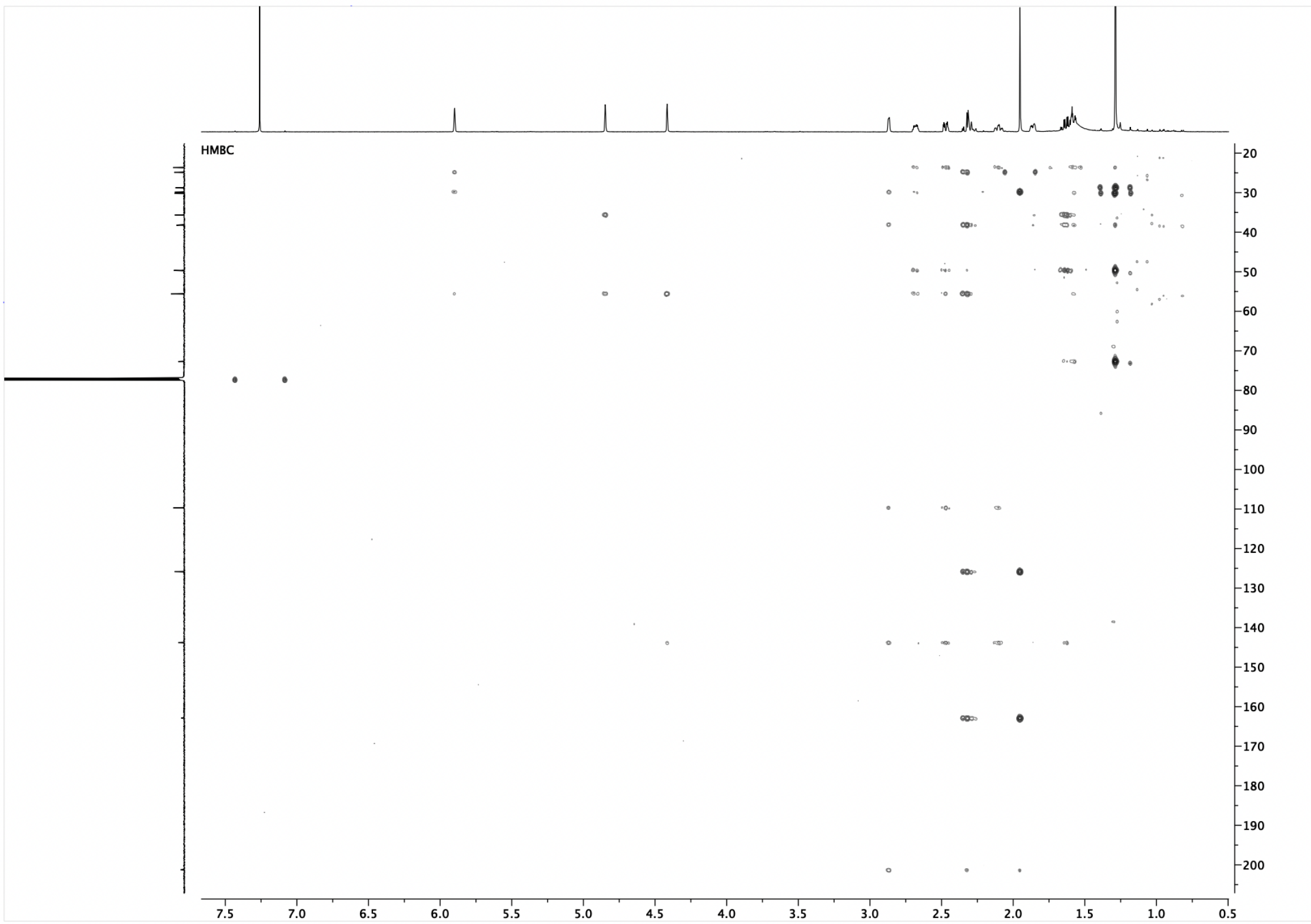

Figure S14. The HMBC Spectrum of Compound **2** in $CDCl_3$

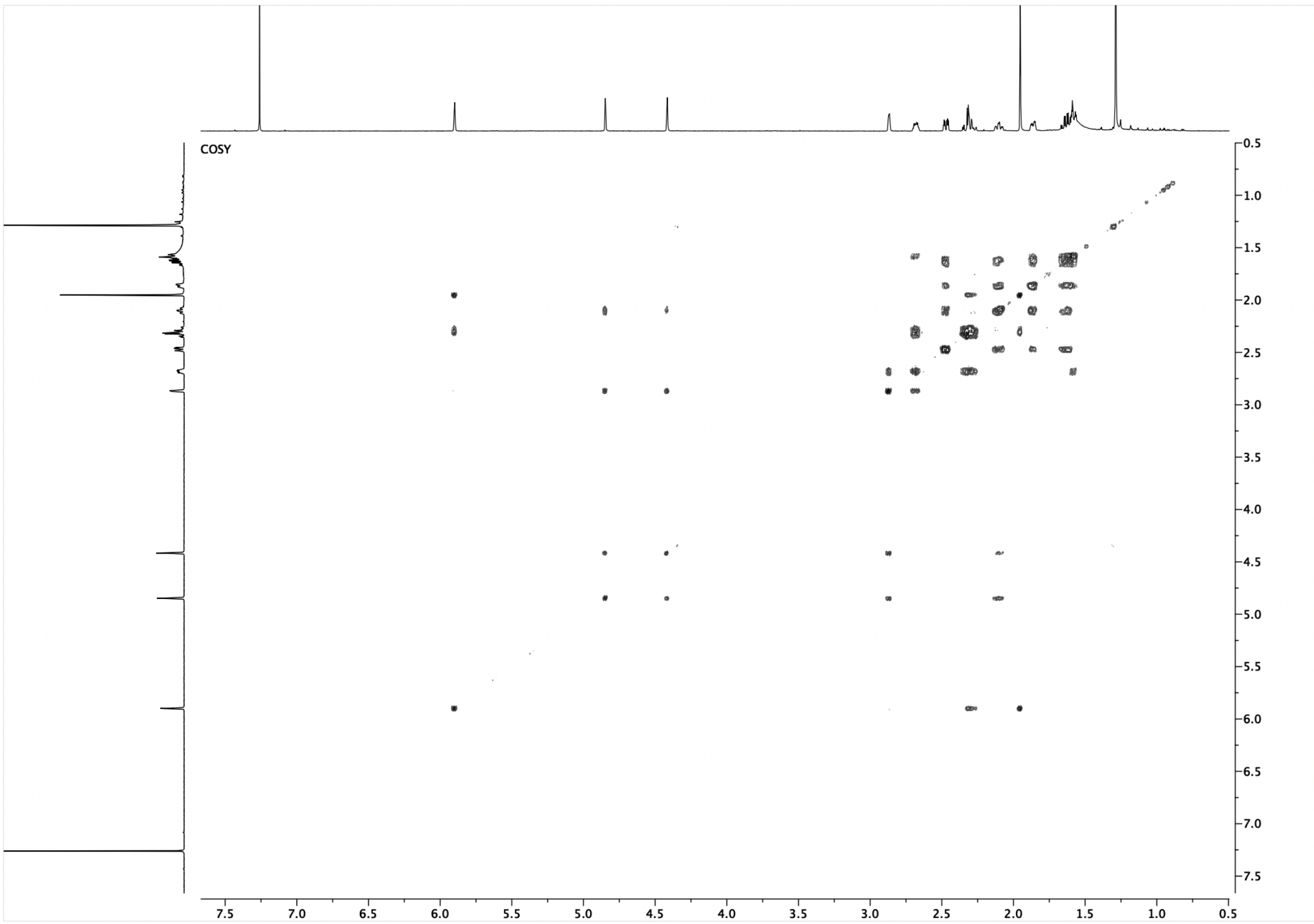

Figure S15. The ^{1}H-^{1}H COSY Spectrum of Compound **2** in $CDCl_3$

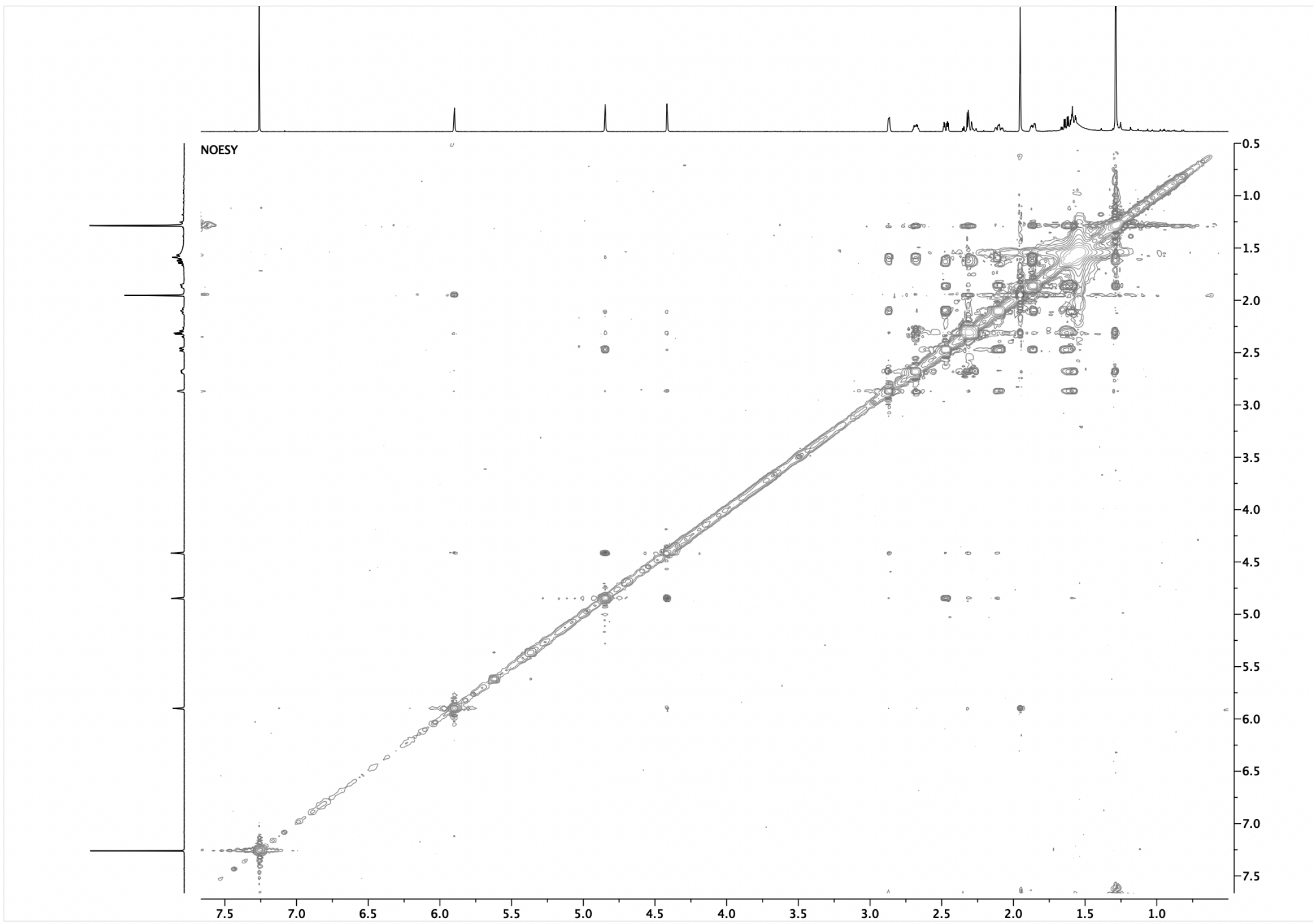

Figure S16. The NOESY Spectrum of Compound **2** in $CDCl_3$
